# Deplete and repeat: microglial CSF1R inhibition and traumatic brain injury

**DOI:** 10.3389/fncel.2024.1352790

**Published:** 2024-02-21

**Authors:** Rebecca Boland, Olga N. Kokiko-Cochran

**Affiliations:** Department of Neuroscience, College of Medicine, Chronic Brain Injury Program, Institute for Behavioral Medicine Research, The Ohio State University, Columbus, OH, United States

**Keywords:** microglia, traumatic brain injury, CSF1R, neuroinflammation, PLX5622

## Abstract

Traumatic brain injury (TBI) is a public health burden affecting millions of people. Sustained neuroinflammation after TBI is often associated with poor outcome. As a result, increased attention has been placed on the role of immune cells in post-injury recovery. Microglia are highly dynamic after TBI and play a key role in the post-injury neuroinflammatory response. Therefore, microglia represent a malleable post-injury target that could substantially influence long-term outcome after TBI. This review highlights the cell specific role of microglia in TBI pathophysiology. Microglia have been manipulated via genetic deletion, drug inhibition, and pharmacological depletion in various pre-clinical TBI models. Notably, colony stimulating factor 1 (CSF1) and its receptor (CSF1R) have gained much traction in recent years as a pharmacological target on microglia. CSF1R is a transmembrane tyrosine kinase receptor that is essential for microglia proliferation, differentiation, and survival. Small molecule inhibitors targeting CSF1R result in a swift and effective depletion of microglia in rodents. Moreover, discontinuation of the inhibitors is sufficient for microglia repopulation. Attention is placed on summarizing studies that incorporate CSF1R inhibition of microglia. Indeed, microglia depletion affects multiple aspects of TBI pathophysiology, including neuroinflammation, oxidative stress, and functional recovery with measurable influence on astrocytes, peripheral immune cells, and neurons. Taken together, the data highlight an important role for microglia in sustaining neuroinflammation and increasing risk of oxidative stress, which lends to neuronal damage and behavioral deficits chronically after TBI. Ultimately, the insights gained from CSF1R depletion of microglia are critical for understanding the temporospatial role that microglia develop in mediating TBI pathophysiology and recovery.

## 1 Introduction

Globally, an estimated 69 million people suffer from traumatic brain injury (TBI) each year (Dewan et al., 2019). In the United States alone TBI affects approximately 2.8 million people per year. This includes over 200,000 hospitalizations and over 50,000 TBI-related deaths (Taylor et al., 2017). In fact, TBI accounts for over 30% of all injury related deaths (Maas et al., 2017). However, mild cases of TBI are often untreated and therefore unreported (Haarbauer-Krupa et al., 2021). As a result, TBI is recognized as a major public health concern and a growing healthcare burden. For example, the US economic burden from TBI is an estimated $76.5 billion annually (Corso et al., 2006). Survivors of TBI are responsible for direct and indirect costs which exceeds medical expenses alone. An equally important consideration is the long-term costs associated with lost earnings, inability to work, and the need for additional supports through social services (Humphreys et al., 2013).

Traumatic brain injury occurs in all age groups, but the predominant causes of TBI vary across the lifespan. Young children (ages 0–4) and the elderly (over the age of 75) have the highest incidence of TBI but are especially susceptible to TBI caused by falls (Haarbauer-Krupa et al., 2021). Teens and adults (15–24 years of age) have an increased risk of TBI caused by motor vehicle crashes (Gessel et al., 2007; Haarbauer-Krupa et al., 2021). Children and teens (10–17), especially males, are vulnerable to sports related TBI (Gessel et al., 2007; Sahler and Greenwald, 2012; Haarbauer-Krupa et al., 2021). In recent years, there has been a decrease in TBI related deaths (Ahmed et al., 2017; Taylor et al., 2017). However, there has been a concurrent increase in emergency department visits and hospitalizations associated with TBI (Taylor et al., 2017). While this could reflect an actual increase in TBI incidence, these statistics may also reflect increased public awareness of TBI and its long-term effects. Together, these data highlight the large number of individuals experiencing brain injury and managing long-term recovery. The consequences of TBI are universally debilitating and often include memory impairment and development of various psychiatric disorders. These deficits can present acutely after injury or develop months later (Coronado et al., 2012; Ahmed et al., 2017). If persistent, these behavioral changes negatively affect quality of life in TBI survivors as well as caregivers months to years after injury (Coronado et al., 2012; Ahmed et al., 2017).

Traumatic brain injury is an extremely complex condition with a wide range of outcomes that evolve as a result of primary and secondary injury. Primary injury is a direct result of the mechanical force causing TBI, resulting in axonal damage and shearing and cell death. Secondary injury involves biochemical, metabolic, and neuroinflammatory responses that occur in response to primary mechanical damage (Xiong et al., 2018). Neuroinflammation is a key factor in the brain’s response to injury (Shao et al., 2022). Neuroinflammatory responses are mediated in large part by microglia, the resident innate immune cell of the central nervous system (CNS) (Shao et al., 2022). The acute microglial response to injury is necessary and considered neuroprotective (DiSabato et al., 2016). Microglia clear debris through phagocytosis and produce cytokines and chemokines that allow for some tissue repair (Donat et al., 2017). However, after injury, accumulating data demonstrate that microglia can become primed (Witcher et al., 2015). In response to a secondary immune challenge, these primed microglia become hyperreactive, leading to a maladaptive neuroinflammatory response that is associated with poor outcome(Fenn et al., 2014; Loane et al., 2014; Norden et al., 2015).

This review aims to highlight the cell specific role of microglia in TBI pathophysiology. First, we discuss neuroinflammation after TBI, with a specific focus on the role of microglia in mediating CNS inflammation within preclinical models. Second, we describe methods for manipulating microglia, including genetic deletion, drug inhibition, and depletion models. Notably, use of Colony Stimulating Factor Receptor 1 (CSF1R) inhibitors revolutionized scientists’ ability to deplete and even repopulate microglia in rodents. The CSF1 receptor is integral to microglia maintenance and survival. Thus, inhibiting CSF1R results in a swift and effective depletion of over 90% of microglia in most mouse strains (Spangenberg et al., 2019). There are different types of CSF1R inhibitors, but PLX3397 and PLX5622 are two small molecule CSF1R inhibitors recognized for their high efficacy (Elmore et al., 2014; Green et al., 2020). Therefore, the last section of the review will focus on underlying patterns between published studies utilizing these CSF1R inhibitors in the context of TBI.

## 2 Neuroinflammation and TBI

### 2.1 Preclinical models recapitulate fundamental features of clinical TBI

Traumatic brain injury is defined as an insult to the brain caused by an external mechanical force. Common causes of head injury include falls, motor vehicle accidents, sports, interpersonal violence, or explosive blast. TBI is often classified as mild, moderate, or severe. Mild TBI accounts for nearly 70–90% of all reported TBIs, but severe TBI has the highest association with mortality (Holm et al., 2005; Kennedy et al., 2022). Although only 11% of TBIs are moderate in nature, they account for longer lasting effects than mild TBI, such as cognitive and social deficits(Dewan et al., 2019). Further, moderate TBIs have lower mortality rates than severe TBI, creating a population of people that likely survive decades after injury. This creates an opportunity for other life events to influence outcome and challenge a primed neuroimmune environment. An estimated 5.3 million people live with a TBI-related disability in the US alone(Selassie et al., 2008). In fact, 5 years after TBI, many people stayed the same but some survivors became worse (Hammond et al., 2004). Thus, mild-moderate severity TBI is increasingly important to investigate and will be the focus of this review.

Traumatic brain injury is typically classified as either a focal or diffuse injury. A focal injury occurs when the skull fractures and enters the brain or when a foreign object pierces through the skull. As a result, the damage caused by focal injuries is limited to the area of the disruption (Andriessen et al., 2010). In a diffuse injury, the mechanical force of rapid acceleration-deceleration causes the brain to hit the skull. Diffuse injuries give rise to widespread damage and can affect many brain regions (Andriessen et al., 2010). Damage from diffuse injury includes diffuse axonal and vascular injury, as well as swelling of the brain (Ekmark-Lewén et al., 2013; Jha et al., 2019; Wu et al., 2021). However, it is worth noting that focal injuries often cause diffuse damage over time (Dixon et al., 1999; Loane et al., 2014).

Clinically, TBI does not occur with isolated characteristics of focal or diffuse injury. Therefore, preclinical models with mixed focal and diffuse characteristics have great translational potential. Critically, no preclinical model captures all aspects of human head injury. However, use of these well characterized models allows investigators to dial in on key aspects of TBI pathophysiology that are translationally relevant. Some commonly used preclinical models of TBI include controlled cortical impact (CCI), fluid percussion injury (FPI), weight-drop injury, primary blast injury, and the Closed-Head Impact Model of Engineered Rotational Acceleration (CHIMERA) (Xiong et al., 2013). CCI models of TBI include use of a mechanical piston that directly impacts the intact dura. This injury results in cortical tissue loss and axonal injury, thus representing key characteristics of a focal injury (Osier and Dixon, 2016; Mohamed et al., 2021). Fluid percussion models of TBI produce a diffuse injury in animal model by inflicting a direct fluid pulse onto the intact dura (Dixon et al., 1987; Alder et al., 2011). This injury results in swelling and subcortical neuronal loss, mirroring clinical characteristics of a mixed focal and diffuse injury. Both CCI and fluid percussion models of TBI require surgical preparation in which a small piece of the skull is removed. This caveat facilitated development of closed head models of TBI, such as CHIMERA and weight-drop, which do not require skull removal and produce a more diffuse injury (Kalish and Whalen, 2016; McNamara et al., 2020). In fact, CCI is now used as either an open or closed headed model in many studies (Shitaka et al., 2011; Osier and Dixon, 2016; Doran et al., 2019). Notably, animals are not placed in a stereotaxic frame for CHIMERA, weight drop, or some blast models of TBI. Therefore, these models capture the effects of head rotation and acceleration/deceleration at the time of injury (Namjoshi et al., 2016; Shishido et al., 2019). Temporal pathophysiology can vary between pre-clinical models of TBI. Nonetheless, the neuroinflammatory response is conserved as a signature of long-term pathophysiology (Simon et al., 2017).

### 2.2 Neuroinflammation drives secondary injury after TBI

Primary injury occurs as a direct result of the external mechanical trauma to the head. Thus, these consequences arise directly after injury and occur within minutes of the initial insult. As a result, potential for therapeutic intervention is limited. Primary injury is predominantly characterized by neuronal death and tissue loss, but also includes contusion, hematoma, and hemorrhage (Mckee and Daneshvar, 2015). Damaged ion channels and an influx of intracellular ions lead to cell death that is considered irreversible (Faden et al., 1989). Further, rotational forces from the mechanical trauma can cause axons to twist, tear, and shear, leading to axonal injury. Axonal injury results in broad disruption in neuronal signaling and thus contributes to acute cognitive deficits (Creed et al., 2011). Cell death and damage results in disruption of the blood-brain barrier, which lends to prolonged deficits from injury (Alam et al., 2020; Cash and Theus, 2020).

Following this acute damage is the secondary phase of injury, in which a more progressive and chronic response is enacted through various biochemical processes within the central and peripheral nervous systems. Secondary sequalae can persist for months to years after injury and include neurometabolic deficits such as increased oxidative stress, prolonged cellular death, and persistent blood-brain-barrier disruption resulting in widespread neuroinflammation (Chen et al., 2016; Wang et al., 2017; Xiong et al., 2021). Increased post-injury oxygen consumption results in oxidative damage and mitochondrial dysfunction leads to depleted energy stores and increased apoptosis (Fesharaki-Zadeh, 2022). Further, the continuous release of excitatory neurotransmitters such as glutamate results in excitotoxicity and the spread of cell death beyond the site of injury (Faden et al., 1989). Injured cells from both primary and secondary injury release cytoplasmic and nuclear proteins that act as damage associated molecular patterns (DAMPs) which trigger immune responses (Zhang et al., 2010; Balança et al., 2021).

Secondary injury is largely mediated by neuroinflammation, which includes increased production of pro and anti-inflammatory cytokines and chemokines (DiSabato et al., 2016). The post-injury neuroinflammatory response is driven by reactive microglia, and other cells such as astrocytes, and infiltrating peripheral immune cells can propagate neuroinflammation over time (Xiong et al., 2018). Ultimately, unchecked or persistent neuroinflammation has the potential to negatively impact neurons and contribute to functional impairment. Several excellent reviews are available that describe post-injury neuroinflammation in detail (Kumar and Loane, 2012; Mckee and Daneshvar, 2015; Chiu et al., 2016; Simon et al., 2017; Xiong et al., 2018). Here, we will provide a brief overview of microglia function as well as a summary of post-injury microglia interactions with other cell types.

## 3 Microglia in TBI

Microglia are derived from a yolk sac progenitor and migrate to the CNS during early embryonic development (Ginhoux et al., 2013). These progenitors continue to proliferate up to 2 weeks postnatally in mice (Alliot et al., 1999). Similarly, microglia like- cells have been found as early as 3 weeks in humans but continue to proliferate throughout gestation (Hutchins et al., 1990; Rezaie et al., 1999). After the developmental period, microglia are long-lasting cells that have relatively low turnover in both rodents and humans (Réu et al., 2017). Microglia make up approximately 10% of the cells within the mature murine CNS (Ginhoux et al., 2013).

Microglia are highly ramified, mobile cells that survey their environment in homeostatic conditions. Microglia have the unique ability to scan the brain’s parenchyma using their branches, which allows them to inspect the CNS for damage and debris. When microglia sense signals from their environment, they undergo both phenotypic and transcriptional changes (Cao et al., 2012). For example, microglia undergo morphological restructuring in which they retract their branches and enlarge their cell bodies. This change allows them to phagocytose debris to minimize damage to the CNS (Donat et al., 2017). However, microglia also undergo a complex change in gene expression in response to signals from their environment. These gene changes often are tailored to the type of immune stimulus. Thus, later discussion of gene expression changes influenced by microglia will be in the context of TBI.

Microglia are highly dynamic and have a wide range of activation states. These activation states are dependent on the context of their environment. Thus, microglia express different genetic markers within the adult CNS, and throughout development, aging, health, or disease. Key markers of microglia identity include *Csf1r*, transcription factor *Pu.1*, transcriptional regulator *Sall1*, cytoplasmic markers including ionized calcium-binding adapter molecule 1 (IBA1), and surface markers such as the purinergic receptor P2Y12, fractalkine receptor (CX3CR1), and transmembrane protein TMEM119 (Paolicelli et al., 2022). Many of these markers are considered homeostatic markers of microglia and can be downregulated with microglia reactivity (Butovsky and Weiner, 2018).

Microglia express a wide variety of receptors that allow them to produce diverse responses to signals in their environment. These include purinergic receptors that respond to ATP (P2X7, P2Y12), neurotransmitter receptors (glutamate, GABA), various cytokine receptors (interleukins, interferons), fractalkine receptor (CX3CR1) and more (Liu et al., 2016; Jurga et al., 2020). This allows microglia to respond to pro-and anti-inflammatory cytokine production from nearby cells such as neurons and astrocytes. Microglia, along with other cell types, release a variety of pro-inflammatory cytokines [e.g., interleukin (IL)-1β, TNF-α, IL-6] and chemokines (e.g., CCL2) (Smith et al., 2012; Kumar et al., 2017). Microglia produce other cytotoxic molecules such as reactive oxygen species and nitric oxide (Dohi et al., 2010; Yauger et al., 2019; Ritzel et al., 2023). Production of these inflammatory factors elicits cellular crosstalk and recruits peripheral cells, further promoting neuroinflammation.

Microglia are considered one of the first centrally mediated responders to TBI. Primary injury results in cellular damage and death. Microglia react to cellular debris and other inflammatory mediators released by damaged cells after TBI. This reactivity entails morphological restructuring, in which microglia become de-ramified with hypertrophic cell bodies and retracted processes (Ferrer et al., 2017). Transcriptomic changes increase production of cytokines, chemokines, and other inflammatory mediators like nitric oxide. For example, damaged cells secrete DAMPs including ATP, mitochondrial DNA, or cytokines that elicit an immune response. DAMPs activate pattern recognition receptors such as toll-like receptors (TLRs) on microglia. TLR activation stimulates pro-inflammatory cytokine production within the microglia within hours after injury (Hua et al., 2011; Zhu et al., 2014; Shao et al., 2022).

Notably, microglia are critical mediators of sexual differentiation in the developing brain (Bordt et al., 2020). There are significant sex differences regarding microglial density and phenotype across many brain regions, including the preoptic area, hippocampus, parietal cortex, and amygdala (Lenz and McCarthy, 2015). Specifically in the preoptic area, a region integral to male sexual behavior, neonatal male rats had increased microglial density that displayed an activated phenotype including hypertrophied cell bodies and decreased processes (Lenz et al., 2013). Although microglia demonstrate comparable transcriptional profiles at embryonic time points, they begin to diverge in early postnatal timepoints (Sullivan and Ciernia, 2022). While these sex differences are essential for sexual differentiation through development, some are maintained through adulthood. For example, there is divergence in cell number and phenotype between sexes during adulthood. Furthermore, microglia in the adult mouse brain have demonstrated sexually dimorphic transcriptional differences. Female mice had increased gene expression in genes relating to morphogenesis, development, and cytoskeleton organization, while male mice had higher expression of genes related to inflammation. In fact, female microglia maintained their female-specific gene expression profile when transplanted into male mice and proved to be neuroprotective against stroke (Villa et al., 2018; VanRyzin et al., 2020).

There are indeed prevalent sex differences in the context of TBI that shape long-term outcome. In clinical populations, males have increased risk for TBI and display a higher death rate after TBI compared to females (Peterson et al., 2022). Nonetheless, multiple studies have suggested that females perform worse on outcome measures, including measures of depression, anxiety, and physical symptoms such as headaches (Caplan et al., 2017; Ma et al., 2019). Despite these differences, males tend to be disproportionately represented in TBI literature (Caplan et al., 2017). As a result, sex as a biological variable requires increased attention in both clinical and preclinical studies.

Preclinical studies of TBI have demonstrate multi-faceted sex differences that impact outcome. In a study of midline FPI (mFPI) in mice, female and male TBI mice differed in cytokine levels, peripheral immune cell populations, and sleep quantity (Saber et al., 2020). Specifically, female mice had higher IL-6 at 1- and 5-days post-injury (DPI), while male mice had more blood and spleen neutrophils at 1, 5, and 7 DPI. Although TBI increased sleep in both sexes, male mice slept more in the first 24 h after TBI (Saber et al., 2020). Another study reported that male mice had increased astrogliosis, neuronal cell death, and lesion volume after CCI injury (1, 3, and 7 DPI), while females did not display these exacerbated outcomes (Villapol et al., 2017). Sex differences are also seen in functional outcomes after TBI. A study using Sprague- Dawley rats investigated both hippocampal dependent (novel object location and Morris Water Maze for spatial working memory) and independent (novel object recognition memory) behaviors at 1- and 4-weeks post CCI injury, as well as anxiety and depressive-like behaviors at 5 weeks post-injury (McCorkle et al., 2022). Male TBI rats had impairments in both hippocampal dependent and independent memory tasks at both 1 and 4 weeks, while female TBI rats had impairments only at the 4-week time point, suggesting a delayed impairment. However, only female TBI rats displayed increased depressive like behaviors in the forced swim task at 5 weeks post-injury (McCorkle et al., 2022). Together, these data demonstrate that sex differences are prominent after TBI across multiple outcome measures.

Given the sexually dimorphic role of microglia in the healthy brain, many investigators have begun to pinpoint microglia as a mediator of sex differences after TBI. One study reported sex differences in microglia/macrophage activation within the first week after CCI injury. Specifically, there was increased Iba-1 positive staining in male mice compared to female mice at 1, 3, and 7 DPI across multiple brain regions including primary somatosensory cortex and the dentate gyrus. Moreover, morphological assessment demonstrated that female TBI mice had microglia that displayed a ramified morphology at 4 h post-injury and 1 DPI, while male TBI mice displayed a more hypertrophied morphology at 1 and 3 DPI. However, females had higher pro-inflammatory cytokine levels (IL-1β) at 4 h post-injury. These findings extended to mRNA levels, in which female TBI mice displayed higher IL-1β and TNFα mRNA expression at 4 h post-injury, while male mice had increased pro-inflammatory mRNA expression at 1 and 3 DPI (Villapol et al., 2017). A study using a cortical stab wound model found similar increases in Iba-1 positive staining at the lesion border in male mice compared to female mice (Acaz-Fonseca et al., 2015). Together, these data demonstrate that microglia may play an integral role in sex differences in TBI outcome. However, the data available is limited and more work needs to be done to fully explore this area of research.

### 3.1 Microglia and astrocytes

Under normal conditions, astrocytes play an integral role in maintaining homeostasis. Astrocytes regulate extracellular potassium levels, water uptake, and blood flow (Sofroniew and Vinters, 2010). Similar to microglia, astrocytes become reactive after TBI. Astrocyte reactivity in the presence of CNS trauma is referred to as astrogliosis, a term encompassing the array of phenotypic and genotypic changes astrocytes make after injury (Michinaga and Koyama, 2021). Astrocytes may react to microglial pro-inflammatory cytokine release. Astrocytes express receptors that respond to inflammatory mediators produced by microglia, including TLRs and IL1R1 (Karve et al., 2016). This can trigger astrocytic production of pro-inflammatory chemokines, including CCL2 (Rodgers et al., 2020). Thus, microglia and astrocytes together contribute to an inflammatory microenvironment within the CNS and may exacerbate each other’s response to trauma.

Reactive astrocytes express more glial fibrillary acidic protein, which is one indication of their activation and proliferation (Wilhelmsson et al., 2004; Singh et al., 2016). Astrocytes can become hypertrophic and increase cytotoxicity due to impaired glutamate transport (Villapol et al., 2014). Microglia contribute to this impaired glutamate buffering by astrocytes (Takaki et al., 2012). Increased pro-inflammatory cytokine production by microglia triggers autocrine signaling which can increase microglial glutamate release (Takeuchi et al., 2006). This glutamate release can inhibit the glutamate transporters present on astrocytes, which results in residual glutamate that can be cytotoxic to nearby cells (Takaki et al., 2012).

Astrocytes are further unique in their maintenance of the blood brain barrier (BBB) along with endothelial cells and pericytes. The distal projections of astrocytes, coined end feet, envelop blood vessels comprised of endothelial cells (Daneman and Prat, 2015). The BBB essentially acts as a border between the central and peripheral nervous systems. The microvasculature that comprises the BBB tightly regulates the movement of ions, molecules, and even cells across the barrier (Daneman and Prat, 2015). As such, the BBB is critical in maintaining CNS health and homeostasis. TBI causes damage to the BBB, which can occur within hours after the initial injury (Cash and Theus, 2020). Microglial and astrocytic signaling contribute to BBB breakdown. For example, release of reactive oxygen species from both cell types disrupts tight junctions and enhance permeability through the BBB (Cash and Theus, 2020). Additionally, microglia can phagocytose astrocytic end feet which compromises BBB integrity (Haruwaka et al., 2019). BBB disruption allows larger molecules, proteins, and cells that would typically be confined to the periphery to invade the CNS.

### 3.2 Microglia and peripheral immune cells

Bidirectional communication between the central and peripheral immune systems increases following post-injury BBB disruption (Alam et al., 2020). Therefore, neuroinflammation is modified by peripheral immune cell infiltration. Microglial contribution to BBB disruption and production of inflammatory mediators, such as pro-inflammatory cytokines and reactive oxygen species are major factors that allow for peripheral immune cell infiltration. Neutrophils are early responding cells and contribute to debris phagocytosis to clear the injury site in the acute stage of injury (Soares et al., 1995; Liu et al., 2018). Neutrophil infiltration and activation can elicit microglia reactivity (Kim et al., 2020). Monocytes are later recruited by cytokine and chemokine release from reactive microglia and astrocytes. Different subsets of monocytes may lead to either tissue repair or damage neurons (Makinde et al., 2017; Chou et al., 2018). Depending on the severity of injury, B and T cells may later be recruited and add to neuroinflammation within the CNS (Alam et al., 2020).

### 3.3 Microglia and neurons

Microglial-neuronal interactions are shaped by both environment and time. Within the healthy brain, microglia and neurons have layered communication that contributes to the survival and development of cells, as well as the activity and connectivity of synapses. Notably, many microglial functions that are considered a distinct feature of developmental processes are sustained throughout adulthood.

Throughout development, microglia play a role in axon guidance and cell survival/death. Both in the embryonic and postnatal brain, microglia are in close proximity to developing axonal tracts in multiple regions including the subpallium, corpus callosum, and hippocampal perforant pathway amongst others (Squarzoni et al., 2014; Rigby et al., 2020). Newer evidence further suggests that microglia contribute to axon guidance by secreting factors that mediate axon growth (Squarzoni, 2015; Rigby et al., 2020). Microglia associate themselves to areas of proliferation in the developing brain. Here, they regulate neural precursor pools by phagocytosing non-apoptotic neural precursor cells, ultimately managing the overall cell number of the brain (Cunningham et al., 2013). *In the adult brain, microglia continue to manage cell death by phagocytosing cells undergoing apoptosis* (Lenz and McCarthy, 2015). In accordance with their roles in regulating overall cell survival and axon outgrowth, microglia contribute to myelin maintenance in both the developing and adult brain. Throughout development, microglia are indispensable mediators of myelination by clearing cellular debris of apoptotic oligodendrocytes, phagocytosing oligodendrocytes and oligodendrocyte precursor cells, and ensuring proper myelin formation by eliminating aberrant myelin (Santos and Fields, 2021; Xu et al., 2023). Microglia are necessary for myelin maintenance in the adult brain as well, specifically by preventing both hypermyelination and demyelination (McNamara et al., 2023).

Microglia-neuronal interactions are essential at the level of the synapse. In development, microglia contribute in large part to synaptic development and maturation. Satellite microglia are known to contribute to synaptic pruning through phagocytosis (Krukowski et al., 2021). Furthermore, microglial secreted factors including IL-10 and brain derived neurotrophic factor help support synapse formation and maturation (Mehl et al., 2022). In the adult brain, microglia continue to contact synapses and survey synaptic connectivity between neurons. They can modify synaptic activity by regulating both long-term potentiation and depression (Lenz and McCarthy, 2015). Additionally, microglia contribute to synaptic maintenance through their interactions with perineuronal nets (PNNs). PNNs are structures within the extracellular matrix that form around a neuron’s soma and proximal processes. They begin to form within development and help to regulate and maintain synapses (Crapser et al., 2021). In the adult brain, microglia are known to regulate baseline PNN form and function through the release of proteases and the phagocytosis of extracellular matrix components. Importantly, microglial secreted matrix degrading enzymes have been linked to loss of PNNs in the diseased brain, which can leave synapses unstable and cause memory impairments (Crapser et al., 2020, 2021).

Within both basal and pathological conditions, neurons express receptors that respond to microglia cytokine production. Neurons express TLRs themselves to respond to secreted DAMPs after injury. Persistent microglia-neuronal signaling can increase inflammation and cause changes in neuronal activity and function. Indeed, studies have shown that after TBI, neurons are initially hypoexcitable within hours after injury, but can become hyperexcitable days after injury (Carron et al., 2016; Ping and Jin, 2016).

Synapses are affected directly after TBI, demonstrated by neuron loss, decreased dendritic spines, and a decrease in post-synaptic density proteins (Wakade et al., 2010; Gao et al., 2011). Furthermore, chronic secondary injury mechanisms including oxidative stress, glutamate excitotoxicity, and neuroinflammation lead to synaptic damage and loss. Microglia may play a key role in this synaptic loss. Hyperreactive microglia in an inflammatory environment are known to increase their phagocytic activity and can engulf synapses. Indeed, satellite microglia are reported after TBI and largely influence synaptic stability (Krukowski et al., 2021). This loss of synapses may contribute to long term cognitive deficits. Thus, neurons are not only affected by both phases of TBI, but also add to the neuroinflammatory environment of the CNS through cellular crosstalk and signaling.

### 3.4 The good and bad of neuroinflammation

Neuroinflammation is not always maladaptive. Neuroinflammatory mechanisms can initially be neuroprotective. For example, after exposure to an infection, microglia can shift their morphology and secrete pro-inflammatory cytokines such as IL-1β that induces adaptive sickness behaviors (Henry et al., 2009; Norden et al., 2014). After injury, some level of neuroinflammation is necessary to protect the brain against further damage. Microglia respond through morphological restructuring and increasing production of pro-inflammatory cytokines and chemokines. This inflammatory phenotype is beneficial acutely, as microglia can clear debris and call upon other immune cells to help. For example, macrophages can assist in phagocytosing dead cells, myelin, and glutamate to foster an environment suitable for recovery (Yu et al., 2022). Anti-inflammatory mediators can also be induced after TBI. For instance, IL-10 and transforming growth factor-β were both upregulated at the RNA level after brain injury (Wang et al., 2013; Divolis et al., 2019). Additionally, TBI induced differential macrophage subsets that have increased arginase expression, and the overexpression of arginase in neurons reduced contusion size after CCI injury (Hsieh et al., 2013; Madan et al., 2018). Thus, there is an established system of checks and balances after injury. This elicits a coordinated response of pro- and anti-inflammatory mediators to simultaneously clear the environment and promote repair that is most efficient acutely after injury. However, sustained neuroinflammation causes widespread damage to the CNS and can impede recovery. While acute microglia activation may help clear debris and prevent neuronal loss, persistent microglial reactivity may exacerbate effects of the primary injury and prolong neuroinflammatory responses after TBI (Loane and Kumar, 2016).

### 3.5 Microglia priming occurs after TBI

Microglia priming can be induced by stress, aging, infection, and TBI (Perry and Holmes, 2014; Witcher et al., 2015; Niraula et al., 2017). For example, clinical studies show that microglia display increased expression of CR3, CD68, MHCII, and PK11195 after single incident and repetitive TBI (Ramlackhansingh et al., 2011; Johnson et al., 2013; Smith et al., 2013; Witcher et al., 2015). Pre-clinical studies describe these CD68+/MHCII+ microglia as being primed and ready for action (Fenn et al., 2014; Loane et al., 2014; Norden et al., 2015). Therefore, primed microglia do not maintain a transcriptional profile that is comparable to a baseline or pre-TBI state. Primed microglia do not actively produce cytokines and chemokines, further distinguishing this intermediate cell profile from chronic neuroinflammation. However, primed microglia are hyperreactive to a secondary immune challenge or stressor and show exaggerated release of inflammatory cytokines and chemokines. This elevated inflammatory profile has persisted for up to 12 months post-injury in a CCI model (Loane et al., 2014). Thus, it is possible for primed microglia to facilitate chronic neuroinflammation as well as cognitive and psychiatric deficits over time.

It is crucial to consider the role of microglial priming in the context of TBI recovery. There are many secondary stressors that TBI survivors face after the initial injury. These include various sleep disturbances, psychological stress (financial burdens, social isolation), and the risk for repeated TBI events. Preclinical studies of TBI have investigated how many different stressors (restraint stress, foot-shock stress, sleep disruption, mechanical sleep fragmentation, early life stress, lipopolysaccharide immune challenge) may synergize to impede recovery from TBI (Tapp et al., 2019). Indeed, experimental models have identified unchecked neuroinflammation and behavioral deficits after post-injury exposure to a secondary stressor. For example, restraint stress after TBI has been found to decrease brain-derived neurotrophic factor, increases BBB leakage, and activated endoplasmic reticulum stress (Griesbach et al., 2012; Gao et al., 2022). Using foot shock as a stressor after a model of repetitive concussive TBI synergized to exacerbate social deficits (10 DPI) and depressive like behavior (18 DPI) (Klemenhagen et al., 2013). Sleep disruption post-injury enhanced microgliosis, astrogliosis, and *Tlr4* expression (Tapp et al., 2020). Moreover, post-injury mechanical sleep fragmentation caused increased expression of genes related to interferon signaling and microglia/macrophage activation, along with microgliosis and deficits in neuronal activation and function (Tapp et al., 2022). Similar neuronal deficits and BBB disruption persisted even after a period of recovery from SF (Tapp et al., 2023). Repeated stress modeling PTSD after TBI altered the mitochondrial electron transport chain and induced behavioral deficits (Xing et al., 2013). Lipopolysaccharide (LPS) is a component of the bacterial cell way and a commonly used tool for peripheral immune challenge. In rats, LPS administration post-weight drop injury increased cytokine production as early as 3 h post-injury and up to 7 DPI (Hang et al., 2004). Injection of LPS after mFPI in mice caused persistent microglial restructuring and increased cytokine/chemokines (IL-1β and TNF-α) at the RNA level chronically (Fenn et al., 2014). Furthermore, LPS immune challenge post-TBI caused chronic cognitive deficits and depressive-like behavior (Fenn et al., 2014; Muccigrosso et al., 2016).

Emerging evidence suggests the timing of stress before TBI may impact outcome. In some cases, pre-injury stress can negatively affect recovery from TBI. Indeed, early life stress before TBI exacerbated memory deficits and cortical atrophy in adulthood (Sanchez et al., 2021). However, all pre-injury immune activating events are not detrimental. The concept of “preconditioning” is that exposure to a less-intense stimulus can be protective against the effects of a subsequent immune challenge or injury (Yokobori et al., 2013; Gao et al., 2021). In the context of TBI, multiple studies have investigated preconditioning by administering LPS before injury. Notably, preconditioning studies and post-injury immune challenges use a similar range of LPS dosages (typically 0.1–0.5 mg/kg). Mice in the preconditioned LPS (0.1 mg/kg) group had less contusion loss, cell death, and reduced neurological deficits up to 1 month post-injury via CCI (Longhi et al., 2011). Another study using the CCI injury model in rats found that preconditioning with LPS (0.1/0.5 mg/kg) 5 days before injury prevented upregulation of IL-1β and TNF-α and reduced neuronal damage in the hippocampus at 4 and 12 h post-injury (Eslami et al., 2015). These findings are consistent across experimental models, as a study using a weight drop model in rats demonstrated that treatment with LPS (0.2 mg/kg) 7 days prior to injury prevented neuronal cell death and glial activation (Turner et al., 2017). Within the context of TBI, careful evaluation of the mechanisms and the timing of preconditioning has yet to be done. Existing studies of preconditioning with LPS before TBI have used a window of 1–7 days before injury to induce the neuroprotective effects of preconditioning. However, it is still unclear if these effects are seen with a longer window of time between LPS administration and injury. While mechanistic investigations in ischemic injury have pointed to the modulation of TLR4 and the downstream modulation of the NF-κB pathway for the neuroprotective preconditioning effects, this has not yet been demonstrated in the context of TBI (Vartanian et al., 2011). Thus, pre-conditioning is an important consideration when discussing pre-injury immune activators. There are many other sources of pre-injury immune stress that have been reviewed elsewhere (Houle and Kokiko-Cochran, 2022). Taken together, these data demonstrate that the synergistic effects of TBI and stress can greatly impact recovery.

## 4 Manipulating microglia in TBI

Cell death and tissue damage that occurs as a direct result from the primary injury is considered irreversible. Thus, the secondary phase of injury, and particularly neuroinflammation, represents an alternative therapeutic target. Microglia are the main mediators of neuroinflammation and can become primed after TBI, leading to chronic, maladaptive responses that impede functional recovery. Thus, microglia represent a highly influential cell type in TBI pathophysiology. To date, microglia have been manipulated through genetic and pharmacologic strategies following TBI.

### 4.1 Genetic manipulation

Genetic methods of microglial manipulation have been utilized to study microglia’s role in development, health, injury, and disease. One of the first transgenic lines using a toxin-based method involved overexpression of the herpes simplex virus-derived thymidine kinase (HSVTK), driven by a myeloid promoter *Cd11b* (Green et al., 2020). Following treatment with ganciclovir, CD11b+ cells undergo apoptosis. Another common toxin model is the diphtheria toxin (DT) approach, in which transgenic mice have the gene for a diphtheria toxin receptor (DTR) that is activated upon Cre recombination (Wieghofer and Prinz, 2016). Transgenic lines with myeloid promoters can be generated to selectively target myeloid lineage cells that will undergo cell death after administration of DT. Unfortunately, the myriad limitations of toxin-based approaches outweigh their benefit as a cell specific tool. While the *CD11b-HSVTK* model has not extensively been used in TBI, a study using CD11b- TK mice in a model of repetitive closed-head TBI induced microglia deletion with increasing doses of valganciclovir, a prodrug for ganciclovir (Bennett and Brody, 2014). The researchers found that at both 7 and 21 DPI, neither low nor intermediate dose microglia depletion attenuated axonal injury. A high dose or sustained treatment with valganciclovir was found to be toxic and increased mortality. Another study exposed CD11b- DTR mice to CCI and found increased pro-inflammatory gene expression in the brain and kidney without changes in lesion size (Frieler et al., 2015). The authors suggest that this inflammatory response could point to a confound of the CD11b-DTR model itself rather than the deletion of macrophages (Frieler et al., 2015). Thus, the use of these approaches is limited temporally, as there is only short-lived depletion and confounds from the sustained use of the toxins.

CX3CR1 is expressed on myeloid lineage cells and dendritic cells. CX3CR1 is highly expressed on microglia, which makes it a viable target for selective microglial manipulation (Wolf et al., 2013). The development of both the heterozygous *Cx3cr1*^*+/GFP*^, *Cx3cr1^*GFP/GFP*^*, and Cx3cr1-/-mice allowed for long-term manipulation of microglia and can be made inducible using tamoxifen using *Cx3cr1*^*+/CreER*^ mice (Wolf et al., 2013; Wieghofer and Prinz, 2016). In models of TBI, there are time dependent effects of Cx3cr1-/- mice. Cx3cr1-/- mice subjected to CCI injury had reduced neuronal death and motor deficits in the acute phase after injury (24 h–15 days) in two separate studies (Febinger et al., 2015; Zanier et al., 2016). Chronically, mice deficient in CX3CR1 had exacerbated neuronal death, cognitive dysfunction, and motor deficits at 30 DPI and 5 weeks post-injury (Febinger et al., 2015; Zanier et al., 2016). There are multiple other microglial specific transgenic lines targeting essential markers such as *Pu.1*, *TGF*-β*1*, or *Irf8*, which result in a complete loss of microglia but also severely impact survival (Basilico et al., 2022). However, these have not been explored in the context of preclinical TBI.

Multiple transgenic lines rely on the colony-stimulating-factor 1 receptor (CSF1R), which is expressed on microglial/macrophage subsets and is essential to survival, proliferation, and differentiation of these cells. The role of CSF1 and its receptor were first characterized using the osteoporotic (op/op) *Csf1^op/op^* mice, which lack CSF1. These mice develop osteoporosis and demonstrate deficits in neuronal outgrowth and connectivity, but only a reduction in microglia, due to sustained IL-34 signaling (Kondo et al., 2007). CSF1R knockout mice were generated to further investigate its importance. The overall lack of this receptor resulted in widespread depletion of microglia along with similar deficits demonstrated by the *Csf1^op/op^* mice (Green et al., 2020). Since then, multiple other transgenic lines targeting CSF1R have been created, including a knockout of a *Csf1r* enhancer the *fms*-intronic regulatory element (FIRE) coined *Csf1r*^Δ *FIRE*Δ *FIRE*^ and *Sall1*^*creER/*+^*Csf1r*^*flox/flox*^ mice, which allows for inducible, microglial-specific knockout of CSF1R (Buttgereit et al., 2016; Rojo et al., 2019; Green et al., 2020). The generation of transgenic lines that manipulate CSF1R were critical for understanding the role of CSF1 and its receptor for microglia/macrophage survival. Mice with genetic manipulations of CSF1R have not been used in studies of TBI. Nonetheless, the information gained from these genetic models could pave the way for the development of pharmacological methods that further advance our knowledge of microglia.

Overall, genetic models of microglia manipulation increased cell type specificity and temporal advantages via inducible targeting or conditional lines. However, genetic approaches are time consuming, require careful control and correction to perfect the model, and may not translate to clinical populations. Thus, while they advance our understanding, they can be viewed as a steppingstone to the development of applicable therapies. Genetic models of microglia have been reviewed more extensively elsewhere (Wieghofer and Prinz, 2016; Green et al., 2020).

### 4.2 Drug inhibition

Another area of interest is inhibiting microglia activation. Prolonged and hyperreactivity to a stimulus pushes microglia into a heightened inflammatory state, where they release cytotoxic and pro-inflammatory mediators that can elicit further damage to nearby cells. Thus, inhibiting microglia after an acute inflammatory state could represent a “sweet spot” for necessary neuroinflammation without chronic deficits. However, it is worth noting that most of the agents used for microglia inhibition are not selectively targeting microglia. Thus, data from these studies should be interpreted with careful consideration.

There are multiple ways to inhibit microglia activation. One of the most common methods of inhibiting microglia is the use of minocycline. Minocycline is an antibiotic that is part of the tetracycline family (Garrido-Mesa et al., 2013). Across different conditions, minocycline has been shown to have anti-inflammatory effects, such as inhibiting pro-inflammatory cytokines, upregulating anti-inflammatory cytokines, and inhibiting caspases to minimize apoptosis (Möller et al., 2016). After TBI, minocycline has demonstrated potential benefits, such as improving learning and memory, decreasing lesion volume, and inhibiting caspase-1 activity (Sanchez Mejia et al., 2001; Lam et al., 2013; Adembri et al., 2014; Simon et al., 2018; Celorrio et al., 2022).

Recent studies have investigated the effects of natural substances on microglia activation. For example, curcumin is a natural compound found in turmeric that is known for its anti-inflammatory properties (Hewlings and Kalman, 2017). Curcumin has been utilized in models of TBI, which have shown that curcumin reduced microglial activation and neuronal apoptosis (Zhu et al., 2014; Farkhondeh et al., 2019). Specifically, curcumin may elicit these neuroprotective outcomes through upregulation of neurotrophic pathways and regulating the TLR-4, nuclear factor kappa B (NF-κB) pathways after TBI (Zhu et al., 2014; Khayatan et al., 2022). Resveratrol is part of the polyphenol group of compounds, and a phytoalexin that is found in grapes, peanuts, and more (Nath et al., 2022). Resveratrol administration after TBI has been found to reduce cognitive deficits, production of pro-inflammatory cytokines, and lesion size (Singleton et al., 2010; Cong et al., 2021). Similar to curcumin, studies suggest that resveratrol is acting through TLR-4 / NF-κB pathways to mediate inflammation (Feng et al., 2016). There are many more potential natural targets for microglia inhibition, including ginsenosides from ginseng and cannabidiol, which have been reviewed elsewhere (Choi et al., 2011; Maurya et al., 2021).

### 4.3 Pharmacological depletion models

Pharmacological depletion models have made a large impact on microglia manipulation strategies. Pharmacological models of microglia depletion began with clodronate containing liposomes (Graykowski and Cudaback, 2021). Clodronate is a drug in the bisphosphonate class that is typically used clinically in the treatment of bone disorders and breast cancer among others (Dando and Wiseman, 2004). Liposomes are small, lipid based artificial vesicles. Liposomes are typically phagocytized by microglia. Thus, when packaged with clodronate, microglia can uptake the clodronate and apoptosis is induced. However, clodronate containing liposomes do not cross the BBB, and thus must be administered through intracerebroventricular injection (Lee et al., 2012; Graykowski and Cudaback, 2021). This method of depletion is not microglia specific, as the liposome can be taken up by other cells that can phagocytose debris, such as macrophages (van Rooijen et al., 1997). Administration of clodronate liposomes activated astrocytes, damaged BBB integrity, and increased pro-inflammatory cytokine production (Han et al., 2019). There are few studies that have investigated the effect of clodronate liposomes in recovery from TBI. One study found that injection of clodronate liposomes before CCI injury increased blood brain barrier permeability and depleted macrophages, monocytes, and microglia in rats (Aertker et al., 2019). Ultimately, although clodronate liposomes can deplete microglia, they foster an inflammatory environment and produce unwanted off-target effects.

Another depletion model utilizes the immunotoxin Mac-1-saporin. Mac1 is the macrophage antigen complex 1, a receptor expressed on myeloid cells including microglia. In this immunotoxin, Mac-1 is coupled to saporin, which induces cell death (Basilico et al., 2022). Thus, when injected intrathecally or via intracerebroventricular administration, Mac-1 targets microglia for depletion. Mac-1-saporin has been used in models of spinal cord injury, ischemic brain injury, and neurodegeneration, but has not been extensively characterized in studies of TBI (Montero et al., 2009; Heldmann et al., 2011; Yao et al., 2016). While mac-1-saporin was effective in depleting microglia across these pathological conditions, this depletion was short-lived and not microglia specific. Moreover, mac-1 saporin had similar off-target effects to clodronate liposomes, including BBB disruption and neuroinflammation (Yao et al., 2016).

These depletion methods helped move the development of pharmacological models forward. Recent identification of effective small molecule inhibitors has reshaped the opportunities and possibilities for microglial depletion. Many of these small molecule inhibitors target the CSF1R receptor, an integral part of microglia functioning. As a result, scientists have taken advantage of these new and improved depletion models to elucidate the role of microglia in the healthy, diseased, and injured brain.

### 4.4 Manipulating colony stimulating factor 1 receptor

Both genetic depletion models and pharmacological depletion methods target the CSF1R. CSF1R is a transmembrane class III receptor tyrosine kinase that is expressed on multiple cell types, including microglia and other myeloid lineage cells such as macrophages and monocytes (Hu et al., 2021). This receptor is also expressed on other cell types including osteoclasts and dendritic cells. Notably, signaling between CSF1R and its ligands [Colony-Stimulating-Factor-1(CSF1) and IL-34] is necessary for the survival, differentiation, and proliferation of microglia (Chitu et al., 2016). These ligands are produced by different cell types. CSF1 in the brain is primarily produced by glia, including astrocytes, oligodendrocytes, and microglia, while IL-34 is primarily produced by neurons (Easley-Neal et al., 2019).

Genetic deletion models have demonstrated that the removal of CSF1 resulted in reduced microglia numbers. However, knockout of the receptor itself led to widespread microglia depletion. Thus, researchers have developed strategies to manipulate microglia via pharmacological methods targeting CSF1R. CSF1R inhibitors are primarily administered through oral routes (Green et al., 2020). Typically, these inhibitors can be formulated into rodent chow. Pharmacological methods mitigate the disadvantages of genetic models with enhanced translational potential. Ki20227, BLZ945, and GW2580 are some of the first CSF1R inhibitors created; however, they lack selectivity. For example, they antagonize CSF1R but also target other receptor tyrosine kinases including cKIT and Fms-like tyrosine kinase 3 (FLT3) (Ohno et al., 2008; Xiang et al., 2023).

Recently developed (and more efficient) CSF1R inhibitors include PLX647, PLX3397, and PLX5622. These CSF1R inhibitors can cross the BBB and have been tested at different concentrations for optimal functioning. To identify the best inhibitor, multiple CSF1R inhibitors were characterized and compared by Elmore et al. (2014). When tested against PLX647 and GW2580, PLX3397 had a lower IC50 value, demonstrating its potency. Additionally, PLX3397 resulted in more effective depletion than other antagonists (Elmore et al., 2014). However, PLX3397 can have off target effects on other receptor tyrosine kinases accompanied by poor brain penetrance (Okojie et al., 2023). Thus, it is not the ideal candidate for microglia depletion. The current gold standard for microglia depletion in mice utilizes PLX5622. PLX5622 is more specific CSF1R and has a higher brain penetrance than other inhibitors, thereby increasing microglia depletion. At the concentration of 1200 ppm within chow, PLX5622 effectively depletes over 80% of microglia within just 3 days of use in C57BL/6 mice (Green et al., 2020). Moreover, PLX5622 has been used in chronic studies, with sustained use for 6 months in studies of Alzheimer’s disease (Spangenberg et al., 2019). This advantage is an important consideration for understanding the temporal aspect of microglia reactivity in health and disease.

Within preclinical studies CSF1R inhibitors have been used to study the role of microglia in neurodegenerative diseases such as Alzheimer’s, Parkinson’s, and Multiple Sclerosis, and injury such as spinal cord injury and TBI among other disease states (Gerber et al., 2018; Oh et al., 2020; Hu et al., 2021). The potential for microglial repopulation is an additional advantage of pharmacological depletion. Discontinuation of CSF1R inhibitor administration allows for microglia to repopulate in the CNS. This repopulation will begin as early as 3 days but takes 14–21 days for full repopulation and return to baseline morphology (Elmore et al., 2014). The origin of repopulated microglia is still under debate. These microglia were initially thought to arise from a unique Nestin+ microglia progenitor within the CNS (Elmore et al., 2015). Recent work by Huang et al. (2018) demonstrated that repopulating microglia may come from proliferation of remaining microglia. This is an ongoing topic of research that will continue to elucidate in years to come. Nonetheless, the ability to repopulate microglia via CSF1R provides an avenue to test key biological questions. Namely, can depletion, repopulation or even “forced turnover” of microglia “reset” the cells back to their baseline state and attenuate inflammation?

## 5 Inhibiting CSF1R on microglia in TBI

We identified 10 published articles that utilized CSF1R antagonists in the context of TBI. Throughout these articles, we identified several underlying themes that connect specific parts of each paper. In reviewing these articles, we will first discuss neuroinflammatory changes that occur with microglia depletion. Next, we will consider how microglia depletion alters oxidative stress and metabolism. We will then examine how other cell types respond to microglia depletion and repopulation, including astrocytes, peripheral immune cells, and neurons. Finally, we will describe different measures of functional recovery that were microglia dependent. Thus, throughout this discussion, articles are summarized within multiple subsections.

### 5.1 Microglia depletion attenuates neuroinflammation

Microglia are the primary propagators of neuroinflammation after TBI. Thus, depletion of microglia has the potential to mitigate the neuroinflammatory environment. Indeed, multiple studies demonstrate that microglia depletion before TBI reduces inflammation. One study investigated microglia depletion before mFPI. At 1 DPI, TBI increased inflammatory gene expression that was partially reversed by PLX5622 administration (Witcher et al., 2021). At this early time point, only 33% of differentially expressed genes were reversed by microglia depletion (Witcher et al., 2021). The authors interpret this to mean that the acute response to injury is microglia independent. Thus, it could be that this early response to injury may depend on other cell types such as fast responding neutrophils. PLX5622 administration before mFPI reduced genes related to inflammation (*Cd68*, *Cd45*, *CcL5*) and signature microglia genes (*P2ry12*) at 7 DPI (Witcher et al., 2018). Complement, interferon, and cytokine/chemokine gene expression were also increased by TBI and reversed by PLX at 7 DPI (Witcher et al., 2018). Similar TBI-induced inflammatory gene expression was increased at 30 DPI, but 80% of differentially expressed genes were microglia dependent, demonstrating that the neuroinflammatory role of microglia evolves over time (Witcher et al., 2021). In a different study that allowed for repopulation of microglia after mFPI, similar increases of inflammatory gene expression (*C1qc*, *Tlr2*) were reversed at 30 DPI (Bray et al., 2022). Thus, forced turnover of microglia can also minimize inflammation and bring microglia back to a homeostatic state.

This attenuation of inflammation is seen in other models of TBI and at other post-injury timepoints. A study by Henry et al. (2020) used a delayed depletion model in which microglia were depleted 4 weeks after CCI injury and repopulated after 1 week of PLX5622 administration (Henry et al., 2020). The authors found that depletion and repopulation of microglia in this chronic phase after TBI resulted in microglial restructuring and reduced inflammatory gene expression (Henry et al., 2020). Interestingly, treatment with PLX5622 decreased TBI-induced NLRP3 inflammasome neuroinflammation as measured by gene expression (*Nlrp3*, *Casp1*, *Il1b*), reduced microglial caspase-1 activity, and IL-1β production at 8 weeks post-injury. Repopulated microglia were ramified compared to TBI induced hypertrophic microglia at 12 weeks post-injury (Henry et al., 2020). Another CCI study depleted microglia after injury (from 1 to 5 DPI) using PLX3397 and then allowed microglia to repopulate until 30 DPI (Wang et al., 2022). After repopulation, there was a reduction in brain tissue loss at the 30 day timepoint. However, female TBI mice displayed higher mRNA expression of inflammatory markers (*Cd68, Casp3*) at 30 DPI compared to male mice (Wang et al., 2022). A study in aged mice (18 months old) that repopulated microglia before TBI showed similar results. Lesion volume after TBI was reduced in the aged brain with microglial repopulation at 2 weeks post-injury. Microglia repopulation in the aged brain also prevented TBI-induced production of pro-inflammatory cytokines such as IL-1β acutely after injury (Ritzel et al., 2023). Overall, microglia depletion and repopulation mediate multiple aspects of neuroinflammation that change over time.

### 5.2 Metabolism and oxidative stress

An emerging area in TBI is the study of energetics metabolism and oxidative stress. After TBI, there is an increased demand for oxygen consumption, which increases the potential for reactive oxygen species production through microglia (Dohi et al., 2010). ROS production can cause mitochondrial dysfunction and oxidative stress (Guo et al., 2013; Fesharaki-Zadeh, 2022). Thus, microglia depletion may prevent neurotoxic microglia from exacerbating neuroinflammation.

One study investigated impaired energy metabolism after TBI by determining lactate concentration in the brain. Lactate buildup after TBI is associated with cognitive decline and higher mortality rates (Lama et al., 2014). Researchers observed increased hyperpolarized [1-13C] lactate-to-pyruvate ratios, demonstrating increased lactate after CCI (Guglielmetti et al., 2017). They observed a decrease in pyruvate dehydrogenase (PDH) as well. PDH is the enzymatic complex that controls if pyruvate can enter the TCA cycle (Lazzarino et al., 2019) This observed decrease in PDH demonstrates the pyruvate is prevented from entering the TCA cycle and thus makes lactate. Pre-injury administration of PLX5622 prevented this increase in lactate-to-pyruvate ratio and the decrease in pyruvate dehydrogenase activity (Guglielmetti et al., 2017). Thus, energy metabolism after TBI is in part microglial dependent. Another study found that delayed depletion of microglia after CCI led to changes in cortical transcriptional patterns related to oxidative stress. At 8 weeks post-injury NADPH oxidase-related genes (*Cybb*, *Ncf1*) were upregulated by TBI but reversed in TBI mice that received PLX5622 (Henry et al., 2020). Other oxidative stress related genes (*Nme5*, *Gsr*) had increased expression after TBI that was decreased in PLX treatment groups. Another study in aged mice (18 months old) repopulated microglia before TBI. The authors found that TBI induced production of ROS were reduced in mice that received PLX5622 2 weeks post-injury (Ritzel et al., 2023).

Together, these findings suggest that energy metabolism and oxidative stress after TBI are dependent on microglia. Evidence of decreased PDH activity and increased lactate ratios indicate mitochondrial dysfunction, which can be caused by microglial ROS production and oxidative stress. These studies show that microglia depletion and repopulation can minimize threat of oxidative stress after TBI in both young and old mice.

### 5.3 Microglia depletion and astrocytes

As previously mentioned, microglia and astrocytes demonstrate dynamic signaling after TBI. Microglia are early responders within the CNS. Their release of pro-inflammatory cytokines can trigger astrocytes to adopt an inflammatory profile. Reactive astrocytes increase their expression of GFAP and become hypertrophied (Wilhelmsson et al., 2006). If astrocytes are responding to inflammation mediated by microglia, microglia depletion may inhibit astrocyte immunoreactivity or provoke a compensatory response.

Multiple studies investigated the response of astrocytes to microglia depletion. Together the data suggests that the contribution of microglia-astrocyte signaling in the response to brain injury can be time and model dependent. For example, in a study of diffuse mFPI TBI increased astrocyte reactivity through increased GFAP percent area at 7 DPI (Witcher et al., 2018). Mice that received PLX5622 before mFPI had reduced amounts of GFAP expression (Witcher et al., 2018). A different study investigated cell-specific gene expression in the cortex using single cell RNA sequencing at 7 DPI after pre-mFPI microglia depletion. Astrocytes at 7 DPI had increased inflammatory gene expression (*Gfap*) which was reversed with microglia depletion (Witcher et al., 2021). Within the same study, cortical neuropathology was examined at 30 DPI, a time point considered to show the chronic effects of TBI. At 30 DPI, TBI–PLX animals had higher mRNA expression of astrocyte-related genes, including *Aldh1l1*, *Gfap*, and Aqp4. Moreover, animals in the TBI-PLX group had increased GFAP labeling at 30 DPI compared to TBI-control animals (Witcher et al., 2021). Thus, at more chronic time points astrocytes may compensate for the loss of microglia by adopting an inflammatory profile. A study of forced microglia turnover also found attenuated astrocytic inflammatory genes (*Gfap*, *Aqp4*) 30 dpi (Bray et al., 2022).

However, multiple studies have found no changes in astrocyte markers. In a delayed depletion model mice were given PLX5622 4 weeks after CCI injury to eliminate chronically actively, neurotoxic microglia. Delayed PLX treatment had no effect on *Gfap* expression levels or GFAP immunolabeling 2 months post-injury (Henry et al., 2020). Additionally, another CCI model using PLX3397 after injury found no changes in astrocyte reactivity in response to microglial depletion (Wang et al., 2022).

In summary, these studies show that astrocytes display time-dependent responses to microglia depletion. Acutely after injury (7 DPI), microglia depletion attenuated astrocyte reactivity. This demonstrates that at this time point after injury, astrocytes may be responding to microglia reactivity. Chronically after injury (30 DPI), astrocytes displayed compensatory mechanisms to help injury recovery in the absence of microglia. These astrocytic responses may be model dependent, as two CCI studies found no changes in astrocyte reactivity. Diffuse models of injury such as mFPI may engage other cell types, such as astrocytes, due to the spread of damage across the brain. Meanwhile, damage due to CCI may limit compensatory mechanisms due to the focal nature of the injury.

### 5.4 Effect of CSF1R inhibition on peripheral immune cells

Although CSF1R inhibitors primarily deplete microglia, they can target other peripheral immune cells. Monocyte and macrophage subsets express CSF1R and can be vulnerable to depletion (Lei et al., 2020). Few studies have investigated the peripheral effects of CSF1R inhibition after TBI. Existing literature demonstrates that CSF1R inhibition does indeed affect peripheral immune cells and that these effects are further altered by TBI.

The study by Giordano et al. (2023) was one of the first attempts to define the peripheral response after pre- mFPI microglia depletion. The authors found that pre- injury administration of PLX5622 increased neutrophils in the brain 7 DPI. Furthermore, microglia depletion changed monocyte populations. There were increased Ly6C high and intermediate monocytes in the blood at 1, 3, and 7 DPI while decreased Ly6C low patrolling monocytes (Giordano et al., 2023). The authors did observe peripheral effects of PLX, specifically in the ablation of CD115+ circulating monocytes. Thus, monocyte populations were changed with CSF1R inhibition and were shifted to an inflammatory state in injured animals on the PLX diet. Microglia depletion also elevated proinflammatory cytokines (IL-1β and TNF-α) in the blood while decreasing anti-inflammatory cytokines (IL-10) (Giordano et al., 2023). Taken together, these data demonstrate that the absence of microglia after TBI may elicit a more robust peripheral immune response. This response may attempt to compensate for the loss of microglia debris clearance and pro-inflammatory cytokine production that can be neuroprotective acutely after injury.

Another study briefly looked at peripheral changes after mFPI to determine if depletion and repopulation of microglia using PLX5622 would alter the presence of cells within peripheral tissue. They found no changes in bone marrow or spleen cells (granulocytes, monocytes, t cells, b cells) between control and repopulation groups(Bray et al., 2022). Thus, even if PLX does result in depletion of these cell types, they also undergo repopulation and return to baseline levels at 30 DPI. Moreover, a delayed depletion model after CCI found that CSF1R inhibition had no effect on infiltrating CD45 hi cell numbers at 8 weeks post-injury (Henry et al., 2020). Thus, delayed microglia depletion did not alter leukocyte trafficking after brain injury, which may be expected at this chronic post-injury time point.

In the response to brain injury, microglia and peripheral immune cells communicate in their combined effort to promote recovery. Together, they elicit a coordinated response that is acutely neuroprotective. Without microglia, the peripheral immune response is acutely exacerbated, possibly to assist in the functions that microglia would normally be doing. However, delayed depletion and repopulation models saw no peripheral effects, which may point to a dampened peripheral response later after injury. These data together demonstrate that peripheral immune responses are partially microglial and time dependent.

### 5.5 Microglia depletion and neurons

Microglia and neuron interactions after TBI can increase neuroinflammation within the CNS and result in increased cell death. Multiple studies have investigated the effect of microglial depletion on neuronal survival, function, and connectivity. Single cell sequencing of cortical neurons at 7 DPI demonstrated TBI-induced suppression of long-term potentiation, synaptogenesis, calcium, and dopamine signaling (Witcher et al., 2021). Mice that received mFPI had reduced dendritic complexity, with a reduction in mature spines and an increase in immature spines. Microglia depletion reversed suppression of these pathways and prevented the reduction in dendritic complexity at 7 DPI (Witcher et al., 2021). One study investigated the effect of CSF1R inhibition before diffuse TBI using single nuclei sequencing at 7 DPI (Packer et al., 2023). This study identified multiple subclusters, including deep-layer and upper layer cortical neurons. TBI induced suppression of cortical neuronal homeostasis (reduction in synaptogenesis and CREB signaling). Microglia depletion before TBI was able to reverse approximately 50% of this gene expression across neuronal subclusters (Packer et al., 2023).

Microglia depletion has some effects on neuronal death and injury. PLX3397 depletion after CCI prevented neuronal apoptosis 5 DPI, as detected by TUNEL (Wang et al., 2022). Another measure of neuronal injury can be changes in functionality. At 30 DPI, TBI induced neuronal connectivity deficits measured by compound action potentials. These deficits were reduced by microglia depletion (Witcher et al., 2018). In a different study, the forced turnover of microglia after mFPI also attenuated these same neuronal connectivity deficits at 30 DPI (Bray et al., 2022). However, changes in dendritic remodeling at 30 DPI were not microglial dependent (Bray et al., 2022). In summary, these data demonstrate that microglia-neuronal communication is a key factor in neuronal gene expression, form, and function. These data further point to reactive microglia as a catalyst for disproportionate neuronal inflammatory mechanisms and ultimately, neuronal damage. These chronic neuronal deficits may lend to persistent behavioral changes after injury.

### 5.6 Functional recovery after CSF1R inhibition

Another consideration in pre-clinical studies of TBI is how neuroinflammation can impact functional recovery after TBI. Functional recovery can span multiple facets, including motor impairments, cognitive functioning, and neuropsychiatric measures. Studying these aspects of recovery are necessary for understanding how inflammatory mechanisms influence behavioral deficits.

Multiple studies have assessed the cell specific role of microglia in motor function post-TBI. In two studies of microglial depletion before injury, motor function was not altered by the absence of microglia as measured by rotarod performance (gross motor coordination) and the latency to escape the horizontal bar-hang test (forelimb strength and coordination) at 7 or 30 DPI (Deacon, 2013; Witcher et al., 2018, 2021). However, a study utilizing PLX3397 after CCI injury reported sex-specific alterations in motor function, in which female mice that received treatment with PLX3397 displayed more motor deficits compared to males at 14 DPI (Wang et al., 2022). Additionally, the authors used the Neurological Severity Score to assess neurological deficits including balance, motor ability, and general behavior. The observed female-specific deficits in motor function were accompanied by increased Neurological Severity Scores at 30 DPI that were not present in male mice. Another study using delayed depletion after CCI injury found that TBI-induced deficits in a beam walking test were reversed with microglia repopulation at 8-, 10-, and 12-weeks post-injury (Henry et al., 2020). The beam walk test assesses fine motor coordination which may explain the variation in these studies (Luong et al., 2011).

Other facets of functional recovery include memory and cognitive functioning which can be measured by a plethora of behavioral tasks. A study by Willis et al. (2020) investigated the effect of depletion of microglia before CCI injury on spatial navigation and memory using the active place avoidance task (Bahník and Stuchlík, 2015). The authors found that depletion did not ameliorate spatial learning deficits or hippocampal neurogenesis at 12 DPI (Willis et al., 2020). However, repopulation of microglia reduced spatial learning deficits and allowed for functional neurogenesis in the hippocampus after injury that was dependent on interleukin-6 at 12 DPI (Willis et al., 2020).

Memory tasks can have multiple aspects, including both spatial and non-spatial learning and memory. At 30 DPI, there were TBI-induced memory deficits as measured by the novel object location (NOL) and novel object recognition (NOR) test (Witcher et al., 2021). The NOL measures hippocampal spatial learning while the NOR measures non-spatial hippocampal and cortical memory (Denninger et al., 2018). Microglia depletion before TBI prevented these memory deficits (Witcher et al., 2021). This same prevention of TBI-induced memory deficits as measured with the NOR/NOL task was present after forced turnover of microglia post-injury at 30 DPI (Bray et al., 2022). TBI can also lend to long term development of neuropsychiatric disorders. The study by Bray et al. (2022) found that TBI induced depressive like behavior in mice 30 DPI as measured by the tail suspension test. Forced turnover of microglia attenuated this depressive-like-behavior after injury (Bray et al., 2022).

In a CCI model of delayed depletion, treatment with PLX5622 was able to prevent hippocampal-dependent working memory deficits as measured using a Y maze task at 10 weeks post-injury. In this same model, TBI increased spatial learning and memory deficits as measured through the Morris Water Maze. At 12 weeks post-injury, mice that had undergone microglial repopulation had reduced spatial reference memory deficits (Henry et al., 2020). Mice that had received PLX used more efficient search strategies as compared to TBI mice on a control diet (Henry et al., 2020). Spatial working (Y Maze) and recognition memory (NOR test) were also improved after pre-injury microglia repopulation in the aged brain. Data from these studies support cognitive measures from mFPI studies. Taken together, these data reinforce that microglia play an important role in hippocampal based memory.

## 6 Conclusion, considerations, and future directions

TBI is complex disorder that can result in long term neurological and neuropsychiatric deficits. Secondary injury, driven by neuroinflammation, lends to these maladaptive outcomes long term (Shao et al., 2022). Microglia priming after TBI can exacerbate this neuroinflammatory environment and impede recovery (Loane et al., 2014; Witcher et al., 2015). This review aimed to discuss the microglia specific role in TBI pathophysiology, with a specific focus on microglial influence on post-TBI outcomes and cell-specific responses.

**FIGURE 1:**
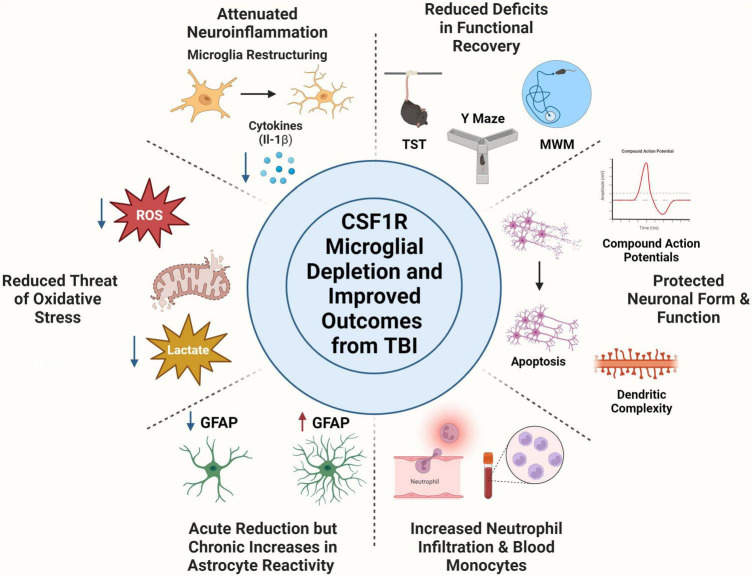
Colony Stimulating Factor 1 Receptor (CSF1R) microglia depletion improves outcome from traumatic brain injury. Microglia depletion attenuated TBI induced neuroinflammation as seen through microglia morphological restructuring and decreased pro-inflammatory cytokine production. Use of CSF1R inhibitors reduced threat of oxidative stress after injury by decreasing reactive oxygen species production and reducing lactate build up post-injury. Microglia depletion had time-dependent effects on astrocytes, in which they undergo an acute reduction in reactivity measured by GFAP expression, but chronically become immunoreactive to compensate for microglial absence. CSF1R inhibition did elicit a peripheral immune response, including neutrophil trafficking to the brain and increases in monocyte population in the blood. Neuronal populations are protected from apoptosis, decreased dendritic complexity, and deficits in compound action potentials with microglia depletion. Microglia depletion rescued multiple behavioral deficits including depressive like behaviors (tail suspension test), spatial working memory (Y Maze), and spatial reference memory amongst others. Created with www.BioRender.com.

Colony stimulating factor 1 receptor inhibition is a valuable research strategy that has helped researchers discover critical roles of microglia in different contexts. Together, the preclinical TBI studies described in this review show that microglia depletion attenuates inflammation acutely and chronically, minimizes threat of oxidative stress, and reverses deficits across multiple facets of functional recovery (motor, memory, and psychiatric measures), Figure 1. Moreover, microglia depletion in TBI studies has revealed possible compensatory mechanisms of astrocytes, shifts in peripheral immune cell populations, and has protective measures on neuronal populations. Forced turnover of microglia in the context of TBI has also demonstrated a return of microglia to a homeostatic-like state, demonstrated through reversal of inflammatory gene expression and restructuring back to a ramified morphology. Although CSF1R is typically considered a microglial target, multiple studies have pointed to effects on other myeloid cell types. Indeed, this has been shown acutely after TBI. CSF1R inhibition almost completely ablated circulating CD115+ monocytes, reduced Ly6cint monocytes in the blood, and reduced neutrophil levels in the brain and blood at 1, 3, and 7 DPI (Giordano et al., 2023). However, the effect of long-term CSF1R inhibition on peripheral cell populations, which demonstrate rapid turnover, remains understudied. Finally, post-injury functional recovery is temporally influenced by microglia with notable influence during the chronic phase of TBI pathophysiology.

Nonetheless, many outstanding questions remain. Notably, people who have suffered from TBI previously are more vulnerable to subsequent TBI in the future (Dams-O’Connor et al., 2013). Repeated TBI is especially common in athletes, victims of physical assaults such as interpartner violence, and military personnel who have received multiple blast injuries (Graham et al., 2014; McKee and Robinson, 2014; Costello and Greenwald, 2022). To date, no repeated models of TBI have investigated the cell specific role of microglia using CSF1R inhibitors. Completing these studies would highlight if microglia impact cumulative effects of head injury. Also, the studies reviewed use adult and aged mice. Thus, future studies can include models of pediatric TBI to determine how CSF1R inhibition affects the microglial response to TBI throughout development. Additionally, more work needs to be done in the space of sex differences. Given that microglia contribute to sex differences in response to injury across multiple preclinical models, future work using CSF1R inhibitors in TBI should investigate sex differences within all outcome measures. A final consideration is determining the relationship between TBI, microglia, and secondary stress. Since microglia priming contributes to exacerbated outcome after post-TBI immune challenge, the use of CSF1R inhibitors may clarify the cell specific role of microglia in this process. Ultimately, pharmacological depletion of microglia using CSF1R inhibition is a beneficial research tool that has helped uncover the dynamic role of microglia within the context of TBI. Still, it is important to consider that CSF1R inhibitors are not a viable therapeutic target for the treatment of TBI at this time. The removal of a major cell population along with depletion of other myeloid cells may negatively impact patient healing. As the effects of CSF1R inhibition on the periphery are poorly understood in humans, there is still much work to be done before this can be considered as a potential therapeutic target for CNS trauma. Future studies will continue to add to our understand of TBI pathophysiology and how microglia shape the landscape of CNS recovery.

## Author contributions

RB: Conceptualization, Writing – original draft, Writing – review and editing. OK-C: Conceptualization, Writing – review and editing.
